# Segmental histomorphometry of the porcine ureter for use as a vascular xenograft

**DOI:** 10.1590/acb397524

**Published:** 2024-10-07

**Authors:** Júlia Galian Ribeiro Táboas, Vivian Alves Pereira da Silva, Marco Aurélio Pereira Sampaio, Aline D’Avila Pereira, Maurício Alves Chagas, Marcelo Abidu Figueiredo

**Affiliations:** 1 Universidade Federal Fluminense – Departamento de Morfologia – Niterói (RJ) – Brazil.; 2 Universidade de Vassouras – Maricá (RJ) – Brazil.; 3 Universidade Federal Rural do Rio de Janeiro – Departamento de Anatomia Animal e Humana – Rio de Janeiro (RJ) – Brazil.

**Keywords:** Heterografts, Ureter, Collagen, Immunohistochemistry

## Abstract

**Purpose:**

To histologically quantify the different tissues that make up the porcine ureter, (epithelial, connective, and muscular tissue) in the three segments labelled: cranial, middle and caudal, in order to identify the segment most compatible for use as a vascular graft.

**Methods:**

Fifteen porcine ureters were collected, divided into the three segments, and the samples were stained with hematoxylin and eosin, picrosirius red and Weigert’s resorcin-fuchsin. The immunohistochemistry technique was applied for alpha-smooth muscle actin. Collagen fibers, muscle, epithelium, and elastic fibers tissue were quantified, in the entire ureter, and divided into hemispheres, comparing the different segments.

**Results:**

When comparing hemisphere segments, significant differences were observed (*p* < 0.01) for collagen and muscle tissue, with the cranial segment presenting the greatest amount of these components when compared to the middle and caudal. No significant difference was observed between the segments when comparing the entire ureters.

**Conclusions:**

After comparing the segments by hemisphere, the cranial segment presented a slight advantage for use as a vascular graft due to presenting greater collagen fiber content.

## Introduction

Vascular grafts are biological or synthetic structures capable of replacing an occluded or damaged vessel^1^. They are necessary in trauma cases, mainly due to cardiovascular diseases, which are the main cause of death world-wide^1^ and for use as an access route in dialysis, given the growing number of kidney failure patients on transplant waiting lists^2^.

Generally, autologous grafts are the first choice, when a patient’s vessel is used to replace a damaged one. The main problem associated with this method is pre-existing pathological conditions, such as diabetes, which can lead to fragile vessels and difficulty in healing. Furthermore, there is greater trauma for the individual involved in these surgeries^3^. Synthetic grafts are also an option, but they are associated with higher infection rates when compared to autologous grafts^4^, as well as problems associated with thrombosis, especially in smaller caliber vessels^3^.

Therefore, xenografts are a promising alternative to synthetic and autologous vascular grafts, and are prepared using tissue bioengineering techniques, employing biological models from species other than that of the recipient^5^. One of the preparation techniques is decellularization, in which a platform called a scaffold is created to guide cell growth in the host. To do this, all cellular components are removed, leaving only an extracellular matrix that is formed by different types of collagens, glycoproteins such as laminin and fibronectin, glycosaminoglycans, and signaling molecules, among others^6^. The composition and architecture of the tissue is maintained, providing adhesion sites, which facilitate adhesion, heterogeneity, and cell separation in the host^7^. An ideal scaffold should resemble the relevant tissue as closely as possible, maintain its shape to fill the defective site, be easily implantable, enhance tissue regeneration, and provide tissue-specific functions^8^.

The porcine ureter is a model with large-scale production capacity which presents important characteristics for scaffold functioning, including a conical tube formation capable of simulating blood vessels^9^. It has a wall made up of smooth muscle fibers arranged in a helical arrangement, in addition to the presence of collagen fibers that provide rigidity to the wall^10^. It has already been anatomically described that the ureter is divided into three segments^11^: cranial (upper), located between the renal pelvis and the upper edge of the bony pelvis; middle (intermediate portion); and caudal (lower), which opens in the bladder triangle^12^. Studies on the histological differences of these portions are necessary in order to assess whether these portions have homogeneous tissue characteristics or whether there are morphological variations that could influence their biocompatibility.

This study sought to histologically quantify the different tissues that make up the porcine ureter, comparing epithelial, connective, and muscular tissue in the three segments: cranial, middle, and caudal, in order to identify the segment most compatible for use as a vascular graft.

## Methods

### Sample collection

Fifteen porcine ureters were collected, all from adult animals, from a homogeneous batch on the slaughter line at the Fripai Alimentos slaughterhouse, located in Juiz de Fora, Minas Gerais, Brazil. As these are samples from commercial slaughterhouses, there was no need to submit them to Animal Use Ethics Committee.

The ureters were collected from the same slaughter line, with the interval of 1 hour between the first ureter and the last one. They were cut into two regions: one close to the renal pelvis, and the other close to the vesicoureteral junction, identified by tying with cotton threads.

### Histological processing and stains

The collected materials were immediately fixed in a 10% buffered formaldehyde solution. Subsequently, each ureter was divided into three segments of similar length, one cranial (close to the kidney), one medium, and one caudal (close to the bladder). A 2-cm thick fragment was removed from the median portion of each ureter segment, which was processed using the standard paraffin technique. The blocks obtained were cut using a Leica 2125RT microtome, producing 5-µm thick sections.

Preliminary tissue analysis to evaluate specimen integrity and morphology was carried out using microscopy analysis with hematoxylin-eosin (HE) staining. From this, sections from each block were processed using Masson’s Trichrome, picrosirius red, Weigert’s Fuchsin-Resorcin techniques, in addition to separating a slide to perform immunohistochemistry for smooth muscle alpha actin.

### Immunohistochemistry technique

Immunohistochemistry was performed to confirm smooth muscle fibers. Sections were deparaffinized in xylene, hydrated through a descending series of ethanol and water, and washed in phosphate-buffered saline (PBS) for 5 min. Subsequently, the sections were treated at room temperature using a 3% hydrogen peroxide solution in methanol to inhibit endogenous peroxidase activity. The sections were then washed in PBS (3 × 5 min) and incubated with 1% goat serum in a humidified chamber for 30 min at 37°C. Subsequently, the sections were incubated with anti-alpha-actin antibody (1:400, A-2547, Sigma-Aldrich Co, St. Louis, MO, United States of America) in a humidified chamber overnight. Negative controls were incubated with PBS instead of the primary antibody. Sections were washed in PBS (3 × 5 min) and incubated with EnV + Dual Link System HRP polymer (Dako; K4061; United States of America) in a humidified chamber for 30 min at room temperature. Finally, slides were developed using a reaction with 3,3’-diaminobenzidine (DAB) at room temperature before being dehydrated and mounted with Entellan (Merck, Darmstadt, Germany)13. Finally, images were captured for quantitative analysis, using a Leica DM500 optical microscope, coupled to a Leica ICC50 HD digital camera.

### Morphometry

The morphometric tissue analysis was performed by capturing images of histological sections using an Olympus BX51 microscope, coupled to a digital camera, Olympus DP72, with 40x magnification, to include the entire ureter in a single capture. The sections stained with picrosirius red followed the same technique observed under polarized light and were analyzed only qualitatively.

**Figure 1:**
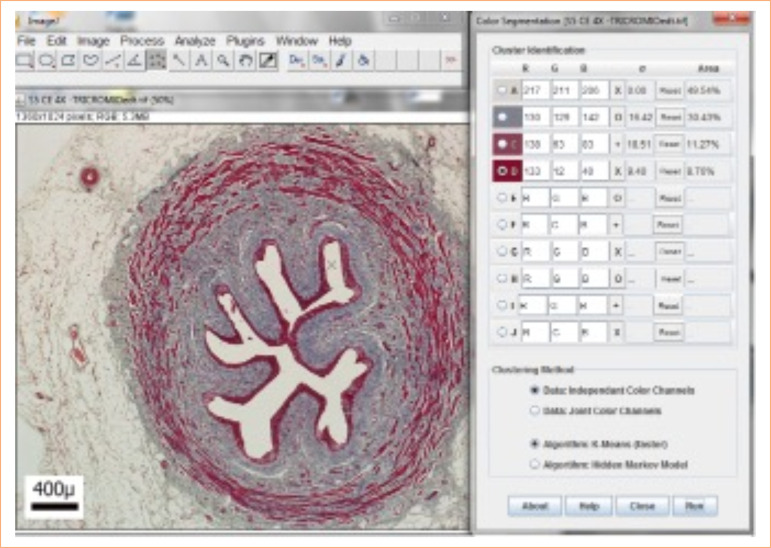
Representation of the color segmentation plugin from the ImageJ software. Porcine ureter Caudal segment. 40x magnification.

Source: Elaborated by the authors.

**Figure 2:**
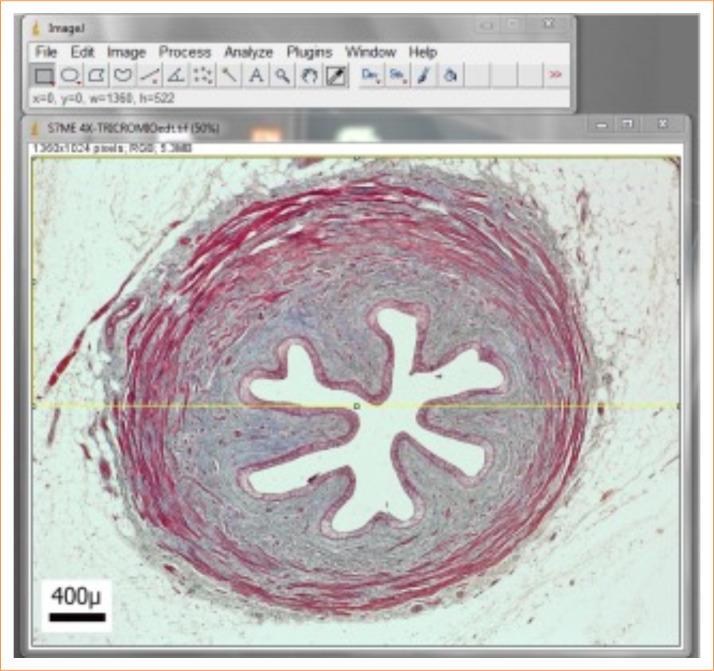
Division of the ureter into two hemispheres, according to differences in smooth muscle arrangement on the wall. Porcine ureter cranial segment. 40x magnification.

Source: Elaborated by the authors.

To analyze the slides quantitatively, ImageJ software was used, with the “color segmentation” plugin, to quantify the types of tissue on each slide (Fig. 1). Two groups of analyses were carried out: one considering the entire surface, and the other separating the image into two hemispheres, using the “rectangle” tool in the ImageJ software and taking the helical distribution of the smooth muscle into account, given its non-homogeneous arrangement on the wall. As such, all ureters were divided into “Hemisphere 1” and “Hemisphere 2”, so that comparisons between different segments could occur from similar hemisphere. Wall and epithelium thickness were also measured for comparison between hemisphere segments, using the “straight” tool of the same program (Fig. 2).

Masson’s trichrome staining was used for quantification, because it facilitates differentiation between collagen fibers that stain blue and muscle fibers that stain red. Furthermore, immunohistochemistry for alpha smooth muscle actin was performed as an additional confirmation method, ensuring the accuracy of the counts. Weigert’s Fuchsin-Resorcin with prior oxidation with ozone was used as it is the technique traditionally used for analyzing elastic fibers^14^. Picrosirius red stain was used to highlight the organization of collagen-containing^15^.

### Statistical analysis

Numerical data were assessed for normality using the Kolmogorov-Smirnov test. Thus, data were presented as mean ± standard deviation, since it is a normal distribution. To compare the segments (cranial, middle, and caudal) according to the tissues (muscle, collagen, and epithelium) of each animal, the repeated measures analysis of variance (ANOVA) test with Tukey’s post-test or the Friedman’s test with Dunn’s post-test, depending on normality, were used. Regarding the comparison between the different quadrants, the univariate ANOVA test with Tukey’s post-test or Kruskal-Wallis’ post-test and Dunn’s test was used, depending on normality. All data were evaluated using the statistical software GraphPad Prism version 8.0, adopting a 5% significance level.

## Results

### Muscle fibers, collagen and epithelium

Table 1 shows data relating to the comparison of muscle tissue, collagen and epithelium by porcine ureter segment. No significant difference between segments was observed.

**Table 1:** Tissue area (mm2) by segment.

	Cranial (n = 14)	Middle (n = 14)	Caudal (n = 14)	*p*-value
Muscle	2,952.2 ± 385.3	3,176.4 ± 843.2	2,869.5 ± 595.3	0.4595
Collagen	5,576.2 ± 423.3	5,280.4 ± 781.3	5,640.2 ± 530.9	0.3126
Epithelium	998.7 ± 248.4	1,002.6 ± 270.2	949.7 ± 207.4	0.7142

Mean ± standard deviation. Repeated measures analysis of variance test with Tukey’s post-test. Source: Elaborated by the authors.

Table 2 presents the comparison of wall thickness and epithelial tissue between the segments, divided by hemisphere. No significant difference was observed.

**Table 2:** Comparison of thickness (mm) between the hemispheres of the different segments.

	Cranial (n = 15)	Middle (n = 15)	Caudal (n = 15)	*p*-value
Wall 1	800.7 ± 245.2	721.2 ± 177.1	814.5 ± 209.4	0.4374
Epithelium 1	57.43 ± 15.86	62.01 ± 18.32	55.26 ± 12.52	0.4934
Wall 2	805.2 ± 222.3	716.0 ± 212.1	723.6 ± 225.4	0.4748
Epithelium 2	55.0 ± 16.1	56.0 ± 12.7	58.5 ± 15.0	0.8018

Mean ± standard deviation. Univariate analysis of variance test with Tukey’s post-test. Source: Elaborated by the authors.

Table 3 shows the data for the comparison of segments by hemisphere, divided by smooth muscle, collagen and epithelial areas. No significant difference was observed.

**Table 3:** Comparison between the area (mm²) of different tissues, between segments based on quadrants.

	Cranial (n = 15)	Middle (n = 15)	Caudal (n = 15)	*p*-value
Muscle 1	1,086 ± 261.1	905.7 ± 270.4	971.7 ± 277.4	0.2061
Muscle 2	1,116 ± 279.2	897.2 ± 258.8	980.2 ± 207.3	0.0709
Collagen 1	2,122 ± 423.8	1732 ± 578.4	1,779 ± 326.4	0.0545
Epitelhium 1	355.6 ± 120.0	324.7 ± 161.0	287.9 ± 85.9	0.3609

Mean ± standard deviation. Univariate analysis of variance test with Tukey’s post-test. Source: elaborated by the authors.

**Figure 3:**
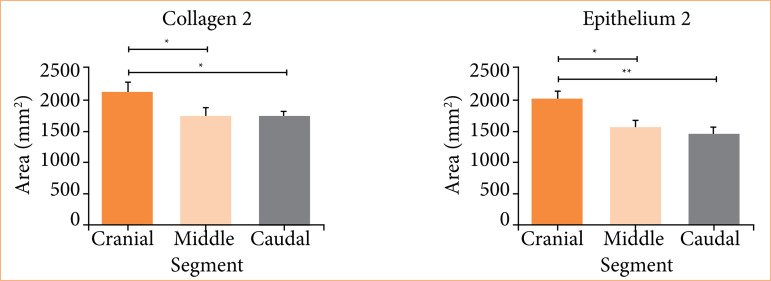
Comparison of tissue areas of different segments by hemisphere (with statistical significance). Univariate analysis of variance test with Tukey’s post-test.

Source: Elaborated by the authors.

Figure 3 presents the comparison between segments by hemisphere. Collagen 2 and epithelium 2 (both referring to the quantification of the respective tissues in the hemisphere of group 2), present a significant difference, in which the cranial segment presented a greater quantity of these tissues in comparison with the middle and caudal segments (collagen 2 at *p* = 0.0222; cranial = 2,145 ± 511; mean = 1,747 ± 458; caudal = 1,736 ± 303; epithelium 2 at *p* = 0.0036; cranial = 411 ± 87; mean = 314 ± 95; caudal = 291 ± 101).

**Figure 4:**
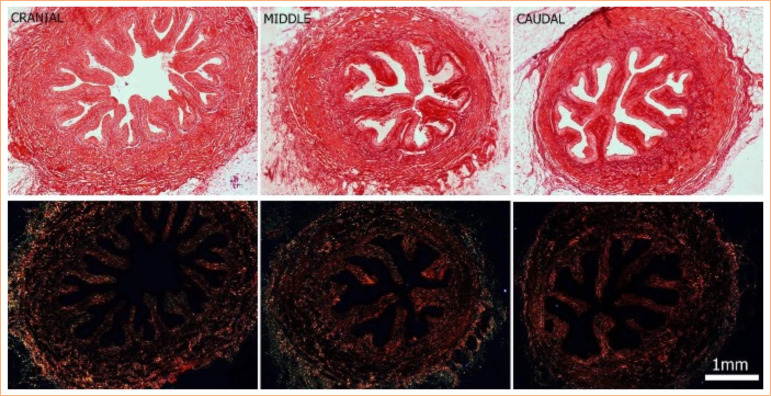
Photomicrograph of the three segments of the porcine ureter: cranial, middle, and caudal stained with picrosirius red. A higher density of collagen type I is seen in all segments, that are from the same sections observed under polarized light. 40x magnification. Scale bar = 1 mm.

Source: Elaborated by the authors.

Figure 4 presents the collagen comparison of the three segments of the porcine ureter by picrosirius red stain. The organization of collagen is homogeneous when comparing cranial, middle, and caudal segments, and the birefringence property of this technique shows that all the segments have an abundant type I collagen (red) and a few thin collagen fibers, such as type III (green).

**Figure 5:**
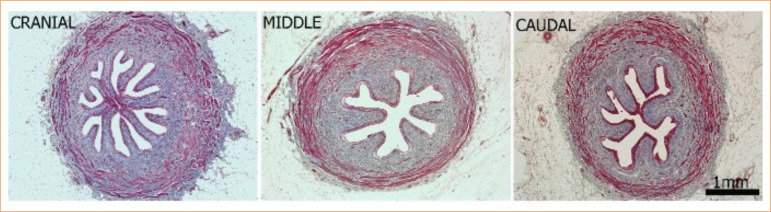
Photomicrograph of the three segments of the porcine ureter: cranial, middle, and caudal stained with Masson’s Trichrome staining. All the three segments had similar quantity of smooth muscle, collagen, and epithelium when using the comparison with entire ureter. 40x magnification. Scale bar = 1 mm.

Source: Elaborated by the authors.

Figure 5 presents the comparison of the cranial, middle, and caudal segments by Masson’s Trichrome staining, emphasizing the collagen quantity (colored by blue) and smooth muscle quantity (colored by red).

### Elastic fibers

**Figure 6:**
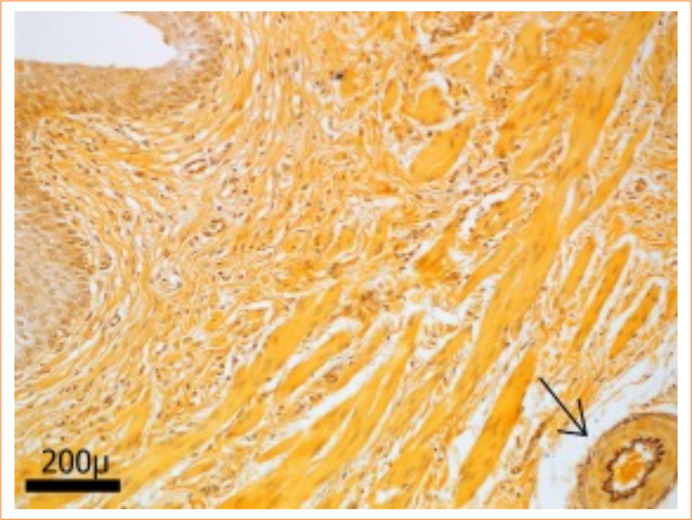
Rare elastic fibers in the middle segment of the porcine ureter. Weigert Fuchsin-Resorcina technique with Orange G arrow demonstrates the blood vessel with evident internal elastic limiting lamina, proving that elastic fibers were stained in the artery wall with the technique, but were scarce in the ureter wall. 200x magnification.

Source: Elaborated by the authors.

Insufficient elastic fibers were found in the three segments for a comparative analysis to be carried out between them (Fig. 6).

### Immunohistochemistry

**Figure 7:**
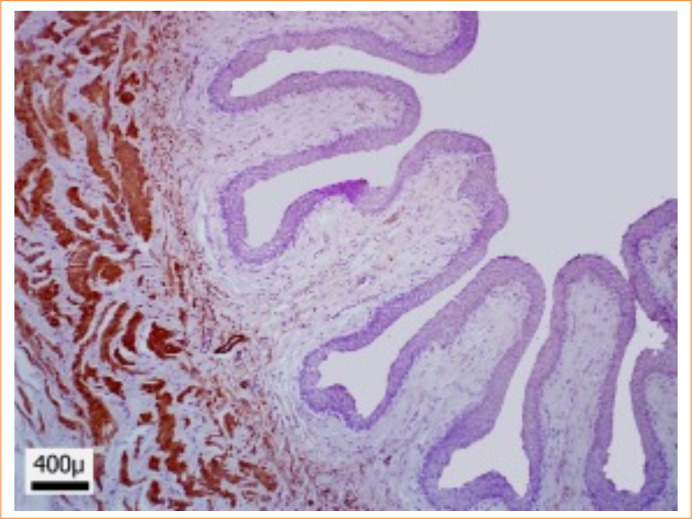
Immunohistochemistry technique for alpha-smooth muscle actin in the cranial segment. 40x magnification.

Source: Elaborated by the authors.

No change was observed for the count in the immunohistochemical analysis for alpha-actin. Therefore, the quantification of smooth muscle made from the distinction of Masson’s trichomere staining was validated (Fig. 7).

## Discussion

The porcine ureter is a potential vascular xenograft model, with large-scale production capacity and compatible anatomical and histological characteristics. Decellularized biological models through tissue engineering allow us to use the tissue’s extracellular matrix and its mechanical properties, and prevent the development of rejection reactions by promoting the removal of cellular waste^1^. This study presents important results for the correlation of histological characteristics of the different ureter segments, thereby contributing to the choice of an ideal graft model.

The area of smooth muscle, epithelial tissue, and collagen fibers was similar in the cranial, middle, and caudal ureter segments. However, there were significant differences when comparing the three segments adopting the hemisphere division model according to the arrangement of smooth muscle in the wall, which causes uneven tissue distribution due to the helical formation.

The cranial segment presented the hemisphere with the highest quantity of collagen fibers. Vascular xenografts manufactured from tissue bioengineering need to have mechanical properties to guarantee integrity of anastomotic sutures and to be able to withstand blood flow pressure without rupturing^16^. Collagen is responsible for tissue rigidity, presenting a capacity to withstand tensile forces when arranged in an organized fashion^17^.

A scaffold is produced retaining only elements of the extracellular matrix, such as collagen, and is therefore an excellent model for producing vascular grafts^18^. Collagen is the most abundant protein in the extracellular matrix and plays an important role in promoting platelet adhesion when damage to the vascular wall occurs. Furthermore, collagen fibers influence the formation of new blood vessels derived from pre-existing vessels, which is an important process in vascular damage^19^. Given this, the higher collagen content allows the graft to activate the coagulation cascade and angiogenesis, thereby combating possible future vascular trauma that may occur in the implant location.

The ureter wall has a collagen distribution composed of 85% type I collagen and 5% type III collagen, representing 90% of all collagen fibers^20^. Immunohistochemistry analysis to differentiate the collagen types was not performed in this study, as evaluation by picrosirius red stain already confirms qualitatively the distribution described. Type IV collagen can be also found in the basement membrane associated with the ureteral epithelium, but in small quantity when comparing to other types of collagen^21^.

Regarding smooth muscle, all segments presented a similar quantity. Smooth muscle plays an important role in scaffolds, being capable of promoting tissue regeneration, with new molecules and cells gradually replacing the location previously occupied by smooth muscle bundles^22^. It is known that smooth muscle is removed during the decellularization process, but signaling molecules that promote cell adhesion are maintained. Therefore, all three segments have the ability to induce cell growth and proliferation in a similar way.

Decellularization process aims to remove most of the cellular waste, leaving only the extracellular matrix, which is composed of collagen. The analysis of the diameter of the ureters would not add information to this study, as this value would change after the removal of waste such as smooth muscle and epithelium by tissue preparation techniques, in order to produce a cellular growth platform.

Regarding elastic fibers, insufficient numbers were found in the ureter wall to carry out quantitative analysis in the three segments. Elastic fibers are important components of connective tissues and organs, associated with distensibility and transport capacity, important properties in blood vessels^20^. As such, collagen fibers are even more necessary to compensate for the reduced number of fibers in the elastic system. Given what was previously proposed, the cranial segment appears to be the most promising for use as a vascular graft.

Epithelial tissue was found in greater quantities in the cranial segment during hemisphere comparison, a characteristic which could imply a greater likelihood of cellular residue. In the context of manufacturing decellularized tissues, this could be disadvantageous, given the need to eliminate cellular residue and stimulus antigen as much as possible^23^. However, to solve this problem, we can combine different decellularization techniques used in tissue bioengineering^24^, which are capable of reducing the antigenic stimulus of the graft as much as possible.

## Conclusion

The cranial segment of the porcine ureter presented some advantages from a morphological point of view to function as a vascular graft produced using the decellularization technique, as it has a similar amount of smooth muscle to the other segments and a greater amount of collagen fibers, which is the most important tissue characteristic when choosing a scaffold. Even though it contains a greater amount of epithelial tissue, the characteristics mentioned above overlap with this problem, since the epithelium can be easily removed using a combination of tissue bioengineering techniques to prepare grafts.

## Data Availability

All data sets were generated or analyzed in the current study.
